# Variation in *VEGFA* and risk of cardiovascular disease in the UK Biobank

**DOI:** 10.3389/fcvm.2023.1240288

**Published:** 2023-11-28

**Authors:** Hongyin Chen, Xingyu Lv, Jinzhao Yang, Zhaojun Chen, Wanning Qiao, Tao Zhou, Yang Zhang

**Affiliations:** ^1^ School of Public Health (Shenzhen), Sun Yat-sen University, Shenzhen, China; ^2^ Guangdong Provincial Key Laboratory of Diabetology, Guangzhou Key Laboratory of Mechanistic and Translational Obesity Research, The Third Affiliated Hospital of Sun Yat-sen University, Guangzhou, China

**Keywords:** *VEGFA* variation, cardiovascular disease, lipid metabolism, genetic risk score, Mendelian randomization

## Abstract

**Background:**

Cardiovascular disease (CVD) is an escalating global health crisis, contributing significantly to worldwide mortality and morbidity. Dyslipidemia stands as a critical risk factor for CVD. Vascular endothelial growth factor A (*VEGFA*) is pivotal in angiogenesis and represents a clinical target for CVD intervention. However, the impact of genetic modulation of *VEGFA* on lipid levels and the subsequent risk of cardiovascular events remains unclear.

**Methods:**

We used LDpred2 to calculate genetic scores for lipid levels based on *VEGFA* variation, serving as instrumental variables to simulate the effect of *VEGFA* inhibitors. We then assessed the associations between genetic risk for lipid levels and CVD risk by conducting One-sample Mendelian randomization.

**Results:**

Our results indicated that low-density lipoprotein cholesterol [LDL-C; odds ratio (OR) = 1.09, 95% CI: 1.06–1.11], remnant cholesterol (RC; OR = 1.24, 95% CI: 1.13–1.36), and triglycerides (TG; OR = 1.14, 95% CI: 1.07–1.22) were positively associated with the incidence of CVD. In contrast, high-density lipoprotein cholesterol (HDL-C) was inversely associated with the incidence of CVD (OR = 0.80, 95% CI: 0.76–0.86). When considering the genetic score for LDL-C constructed based on *VEGFA*, the group with a high genetic score demonstrated an elevated CVD risk (OR = 1.11, 95% CI: 1.04–1.19) compared to those with a low genetic score. Notably, One-sample Mendelian randomization results provided evidence of a causal relationship between LDL-C and CVD (*p *= 8.4×10^−3^) when using genetic variation in *VEGFA* as an instrumental variable.

**Conclusions:**

Genetic variation mimicking the effect of *VEGFA* inhibition, which lowers LDL-C levels, was causally associated with a reduced risk of cardiovascular events. These findings offer insight into the potential therapeutic relevance of modulating *VEGFA*-mediated lipid changes in the prevention and management of CVD.

## 1. Introduction

Cardiovascular disease (CVD) persists as the leading cause of mortality worldwide, exerting a substantial toll on public health and healthcare expenditures (1). Among the myriad risk factors associated with CVD, dyslipidemia stands out as one of the most prevalent. Prolonged exposure to elevated levels of low-density lipoprotein cholesterol (LDL-C) has been shown to escalate the relative risk of CVD mortality by 50%–80% (2). Individuals characterized by primary low high-density lipoprotein cholesterol (HDL-C) are at an elevated risk of developing CVD compared to those exhibiting optimal lipid profiles (3). Notably, low-density lipoprotein-triglyceride (LDL-TG) presents itself as a potential marker for perturbed residual lipoprotein metabolism, significantly correlated with an increased risk of CVD (4). Furthermore, extant research underscores a causal relationship between lipid metabolism-related parameters and CVD (5, 6).

However, the association between various lipid constituents and the risk of CVD continues to exhibit variability due to variances in study populations. For instance, large-scale prospective cohort studies have illuminated that heightened levels of traditional lipid metrics, including total cholesterol (TC), LDL-C, and triglycerides (TG), in conjunction with diminished HDL-C levels, are connected to an augmented risk of CVD (7, 8). Nevertheless, the relationships between TC, HDL-C, LDL-C and the risk of mortality from CVD do not exhibit consistent trends and may manifest a “U” or “J” pattern (9, 10). A Swedish cohort study has even unveiled an association between elevated TC and LDL-C levels and a reduced risk of atrial fibrillation (11). Furthermore, multiple studies have challenged the notion that high LDL-C invariably leads to CVD (12–14). Discrepancies in these findings may be attributed to population-specific variations in lipid profiles across different regions. Moreover, investigations in developed nations have encountered challenges in discerning the adverse effects of small increases in lipid levels on CVD incidence. Consequently, further research is imperative to elucidate the factors contributing to lipid dysregulation and the onset of CVD. Additionally, exploring the potential therapeutic applications of these factors assumes paramount importance.

The vascular endothelial growth factors (VEGFs), a family of secreted signaling polypeptides, play critical roles in stimulating angiogenesis, promoting lymphopoiesis, regulating inflammation, and modulating lipid metabolism. *VEGFA*, the most potent angiogenic-stimulant member of the *VEGF* superfamily, which can be produced by a variety of cell types, including endothelial cells, platelets, macrophages, and tumor cells, has gained significant attention for its crucial role in dynamic homeostasis and pathological processes (15, 16). *VEGFA* stimulates endothelial cell mitosis and migration, enhances microvascular permeability, and promotes angiogenesis by interacting with *VEGF* receptors (VEGFRs) that belong to the tyrosine kinase receptor family (17–19). While it is true that other VEGF family members, such as *VEGFB, VEGFC,* and *VEGFD,* also contribute to the regulation of angiogenesis, lymphangiogenesis, and lipid metabolism (20–24), it's important to emphasize that *VEGFA* remains a crucial focus of research in the context of cardiovascular diseases and lipid regulation. It is reported that the function of *VEGFB* in improving metabolic dysfunction and inducing the browning of white adipose tissue is also dependent on *VEGFA* (25). *VEGFA* can enhance plasma lipids by inhibiting lipoprotein lipase activity (26), and the bioavailability of *VEGFA* is related to intestinal chylomicron absorption (27). Furthermore, *VEGFA* has promising potential as a therapeutic target for CVD. For instance, a novel mRNA-based drug AZD8601, functioning as a “secreted protein” to deliver *VEGFA*, has been developed to promote vascular regeneration and treat heart failure (28). In addition, sequential *VEGFA/S1p* administration with engineered bone marrow (BM) cells improves vascularization and reduces unfavorable cardiac remodeling following myocardial infarction in mice (29). However, the effect of regulating lipid levels by inhibiting *VEGFA* on the reduction of cardiovascular event risk remains unclear.

A shared genetic regulation mechanism may exist between *VEGFA* and cholesterol homeostasis molecules since a common variant highly associated with plasma *VEGFA* levels also contributes to the variation of both LDL-C and HDL-C (30). *VEGFA* expression can be promoted by oxidized low-density lipoprotein (ox-LDL), thereby inducing endothelial dysfunction in human aortic endothelial cells (31). *VEGFA* can influence the change of lipoprotein profiles and increase the proportion of triglyceride in large very low-density lipoprotein (VLDL) particles (26). Although a Mendelian randomization study did not provide substantial evidence to support the positive effect of *VEGF* on ischemic heart disease (IHD), it cannot eliminate the possibility that some specific types of *VEGF* might still have a role in the pathology (32). Angiogenesis, mediated by *VEGFA*, may be involved in plaque instability and thromboembolic events (33). Moreover, *VEGFA* levels are elevated in the serum and plasma of coronary artery disease (CAD) patients (34). Therefore, an accurate assessment of the role of *VEGFA* in CVD is crucial for the precise prevention and management of these diseases. Targeting *VEGFA* in lipid metabolism could be an effective strategy for reducing the risk of cardiovascular events. Our study seeks to leverage individual genetic data to investigate the influence of genetic variation within the *VEGFA* gene on changes in lipid levels and its subsequent impact on susceptibility to CVD. This will provide valuable epidemiological evidence to support the implementation of precision medicine in the context of CVD.

## 2. Materials and methods

### 2.1. Study population

The study cohort comprised 502,469 participants, including 71,318 individuals with cardiovascular events, who were sourced from the UK Biobank (https://www.ukbiobank.ac.uk). The UK Biobank is a comprehensive biomedical database and research resource that provides in-depth phenotypic and genomic data. Participants aged between 40 and 70 years were recruited between 2006 and 2010 from 22 assessment centers in the UK. Data on the participants’ health and genetic information were obtained through questionnaires, interviews, physical measurements, and biospecimen analysis, with all participants providing written informed consent.

### 2.2. Genetic data

The genetic data of the study cohort underwent single nucleotide polymorphism (SNP) genotyping, imputation, and quality control by the UK Biobank team. The first 50,000 participants were genotyped using the Affymetrix UK BiLEVE Axiom chip (35), while the remaining participants were genotyped using the Affymetrix UKB Axiom array (36). Genetic imputation utilized a combined panel of UK10K and 1,000 Genomes phase 3 reference panels. Further information on these processes is available at: http://www.ukbiobank.ac.uk/scientists-3/genetic-data/.

### 2.3. Assessment of CVD

Individuals who have reported experiencing a heart attack, angina, ischemic stroke, or transient ischemic attack (UKB codes 1075, 1074, 1082, 1583 in field 20002; codes 1, 2, 3 in field 6150), undergone cardiovascular procedures (UKB codes 1070, 1071, 1105, 1109, 1095, and 1514 in field 20004), Hospital Episode Statistics database and records of cardiovascular procedures in hospitals (OPCS-4 codes K40-K46, K47.1, K49-K50, K75) and participants who received a hospital diagnosis of CVD (ICD-10 codes G45, I20-I25, I63-I64 or the corresponding ICD-9 codes 410-414, 434, 436 and 42979).

### 2.4. Genetic instruments

LDpred2 method (37), which used summary statistics and a linkage disequilibrium matrix, was utilized to calculate genetic scores (38). This method integrates prior information and genotype data, enhancing the precision of genetic risk prediction. In the analysis of large-scale genetic data, we utilized the R packages “bigstatsr” (https://privefl.github.io/bigstatsr/) and “bigsnpr” (https://privefl.github.io/bigsnpr/).

Firstly, we sourced 7,114 SNPs from the *VEGFA* gene in the NCBI database (https://www.ncbi.nlm.nih.gov/snp/?term=VEGFA) and cross-referenced them with the SNPs derived from the GWAS meta-analysis data concerning HDL-C, LDL-C, remnant cholesterol (RC), total cholesterol (TC), and triglycerides (TG) from the Global Lipids Genetics Consortium (GLGC) (http://csg.sph.umich.edu/willer/public/lipids2013/) (39). We selected the variants that displayed association with the five cholesterol levels (Hardy-Weinberg Equilibrium (HWE) *p*-value >1 × 10^−6^ and minor allele frequency (MAF) >0.01) for further analysis (Supplementary Figure 1). The preliminary analysis of genetic variants in *VEGFA* associated with the levels of these five lipids is presented in Supplementary Tables 1–S5. Subsequently, we utilized LDpred2 to calculate the genetic risk score (GRS) for each participant within the UK Biobank population based on the selected SNPs from each lipid GWAS dataset. The participants were categorized into three groups based on the tertile of their GRS for each of the five lipids (Supplementary Figure 2). Finally, GRS calculated based on *VEGFA* variation was used to evaluate the association between lipid levels and CVD risk.

### 2.5. One-sample Mendelian randomization

One-sample Mendelian randomization (MR) was employed to elucidate the potential causal associations between lipid levels based on *VEGFA* GRS and the risk of CVD. MR is a robust statistical methodology that leverages genetic variation as an instrumental variable to estimate the impact of specific exposure or risk factors on clinically relevant outcomes (40). The framework of MR is illustrated in Supplementary Figure 3. We adopted the Two-stage least squares (TSLS) to perform Mendelian randomization inference in the UK Biobank population. In the first stage, we conducted a regression analysis, wherein the exposure of interest (lipid level) was regressed against the instrumental variable (*VEGFA* variation). This initial stage provided us with the estimated effect of these genetic variants on the exposure, effectively serving as an instrumental variable estimation. In the subsequent second stage, we performed another regression analysis, focusing on the outcome of interest (CVD). We regressed the outcome on the predicted value of the exposure (lipid level), which was derived from the first-stage analysis. The regression coefficient obtained from this stage represented the causal estimate, shedding light on the potential impact of lipid levels on CVD risk, guided by the genetic instrumental variable. A range of covariates for adjustment, including age, sex, ethnicity, body mass index (BMI), cholesterol-lowering medicine, genotyping batch, and PC1-PC10, were made to control for potential confounding factors and enhance the robustness of our causal inference.

### 2.6. Statistical analysis

The statistical analyses were conducted using SAS version 9.4 and R version 4.1.1. Baseline characteristics were reported as means and standard deviations for continuous variables and percentages for categorical variables. Logistic regression was employed to investigate the association between phenotypic lipid profiles and the incidence of CVD and to explore the relationship between lipid levels determined by *VEGFA* genetic variations and CVD risk. The covariates adjusted for in our analyses included age, sex, ethnicity, assessment center, Townsend index, alcohol frequency, smoking status, BMI, and cholesterol-lowering medication. Odds ratios (ORs) with 95% confidence intervals (CIs) were reported to present the results. We applied the Benjaminiand-Hochberg (*BH*) *p*-value correction to account for multiple testing (41). Statistical significance was considered at a 2-sided *α* threshold of *p *< 0.05.

## 3. Results

### 3.1. Participants characteristics

Baseline demographics and characteristics from 502,469 participants [229,117 women (54.41%); mean (SD) age, 56.63 (8.09) years] were analyzed and summarized in Table 1. Among them, 71,318 individuals (14.19%) had pre-existing CVD. The mean (SD) of BMI was 27.43 (4.80) kg/m^2^. The participants’ mean (SD) lipid levels were as follows: LDL-C, 3.56 (0.87) mmol/L; HDL-C, 1.45 (0.38) mmol/L; RC, 1.55 (0.42) mmol/L; TC, 4.56 (0.94) mmol/L; and TG, 1.30 (0.57) mmol/L.

**Table 1:** Baseline characteristics of the population.

	All participants (*N* = 502,469)	Prevalent CVD	
		Yes (*N* = 71,318)	No (*N* = 4,31,151)
Age years	56.63 ± 8.09	60.85 ± 6.65	55.82 ± 8.09
Sex (female%)	229,117 (54.41%)	25,860 (36.26%)	180,232 (57.40%)
Townsend deprivation index	−1.29 ± 3.10	−0.88 ± 3.30	−1.36 ± 3.05
Body mass index^a^, kg/m^2^	27.43 ± 4.80	28.81 ± 5.00	27.21 ± 4.73
Cholesterol medication (Yes, %)	86,878 (17.29%)	34,895 (48.93%)	51,983 (12.06%)
FH (Yes, %)	70,976 (14.09%)	1,960 (2.75%)	11,823 (2.74%)
Ethnicity (%)			
White	472,611 (94.06%)	67,017 (93.97%)	405,594 (94.07%)
Asian	11,452 (2.28%)	2,061 (2.89%)	9,391 (2.18%)
Black	8,058 (1.60%)	885 (1.24%)	7,173 (1.66%)
Mixed	2,954 (0.59%)	312 (0.44%)	2,642 (0.57%)
Other	4,557 (0.91%)	555 (0.78%)	6,406 (0.93%)
Alcohol frequency (%)			
Never	40,627 (8.09%)	7,996 (11.21%)	32,631 (7.57%)
Daily or almost daily	101,753 (20.25%)	14,589 (20.46%)	87,164 (20.22%)
Three or four times a week	115,422 (22.97%)	14,355 (20.13%)	101,067 (23.44%)
Once or twice a week	129,270 (25.73%)	17,308 (24.27%)	111,962 (25.97%)
One to three times a month	55,840 (11.11%)	7,369 (10.33%)	48,471 (11.24%)
Special occasions only	57,996 (11.54%)	9,419 (13.21%)	48,577 (11.27%)
Smoke status (%)			
Never	273,475 (54.42%)	30,539 (42.82%)	242,936 (56.35%)
Previous	173,023 (34.43%)	30,574 (42.87%)	142,449 (33.04%)
Current	52,962 (10.54%)	9,608 (13.47%)	43,354 (10.06%)
Lipids, mmol/L			
LDL-C	3.56 ± 0.87	3.26 ± 0.96	3.60 ± 0.85
HDL-C	1.45 ± 0.38	1.31 ± 0.36	1.47 ± 0.38
RC	1.55 ± 0.42	1.41 ± 0.44	1.57 ± 0.41
TC	4.56 ± 0.94	4.16 ± 1.00	4.63 ± 0.91
TG	1.30 ± 0.57	1.36 ± 0.60	1.28 ± 0.57

Plus-minus values are means ± SD. CVD, Cardiovascular disease; FH, familial hypercholesterolemia; LDL-C, low-density lipoprotein-cholesterol; HDL-C, high-density lipoprotein-cholesterol; RC, remnant cholesterol; TC, total cholesterol; TG, triglycerides.

^a^ The body-mass index is the weight (kilograms) divided by the square of the height (meters).

### 3.2. Associations between phenotypic lipid profiles and CVD

Our observational study in UK Biobank (Table 2) showed that LDL-C, RC, and TG levels were positively associated with the incidence of CVD (OR = 1.09, 95% CI: 1.06–1.11; OR = 1.24, 95% CI: 1.13–1.36; OR = 1.14, 95% CI: 1.07–1.22, respectively Conversely, HDL-C emerged as a protective factor against CVD (OR = 0.80, 95% CI: 0.76–0.86). However, no significant association was found between TC levels and the incidence of CVD in UK Biobank participants (OR = 1.04, 95% CI: 1.00–1.09). The significance of the findings remained unaltered after the application of multiple corrections for the *p*-values. Additionally, after adjusting for the effects of known pathogenic FH variants within the *LDLR, APOB,* and *PCSK9* genes (42), the observational findings remained consistent (Supplementary Table 6).

**Table 2:** Association results between the incidence of CVD and circulating lipid profiles.

	Adjusted demographics^a^		*p*	*p._adjusted_* ^b^
	OR	95% CI		
LDL-C	1.09	1.06–1.11	5.7 × 10^−13^	2.85 × 10^−12^
HDL-C	0.80	0.76–0.86	4.9 × 10^−12^	1.23 × 10^−11^
RC	1.24	1.13–1.36	8.0 × 10^−6^	1.33 × 10^−5^
TC	1.04	1.00–1.09	0.047	4.7 × 10^−2^
TG	1.14	1.07–1.22	2.6 × 10^−5^	3.25 × 10^−5^

LDL-C, low-density lipoprotein-cholesterol; HDL-C, high-density lipoprotein-cholesterol; RC, remnant cholesterol; TC, total cholesterol; TG, triglycerides; OR, odds ratio; Cl, confidence interval.

^a^ Adjusted for age, sex, ethnicity, assessment center, Townsend index, alcohol frequency, smoke status, BMI, and cholesterol-lowering medicine.

^b^ The adjusted *p* was calculated by controlling the *BH*.

### 3.3. *VEGFA* GRS and cardiovascular events

**Figure 1:**
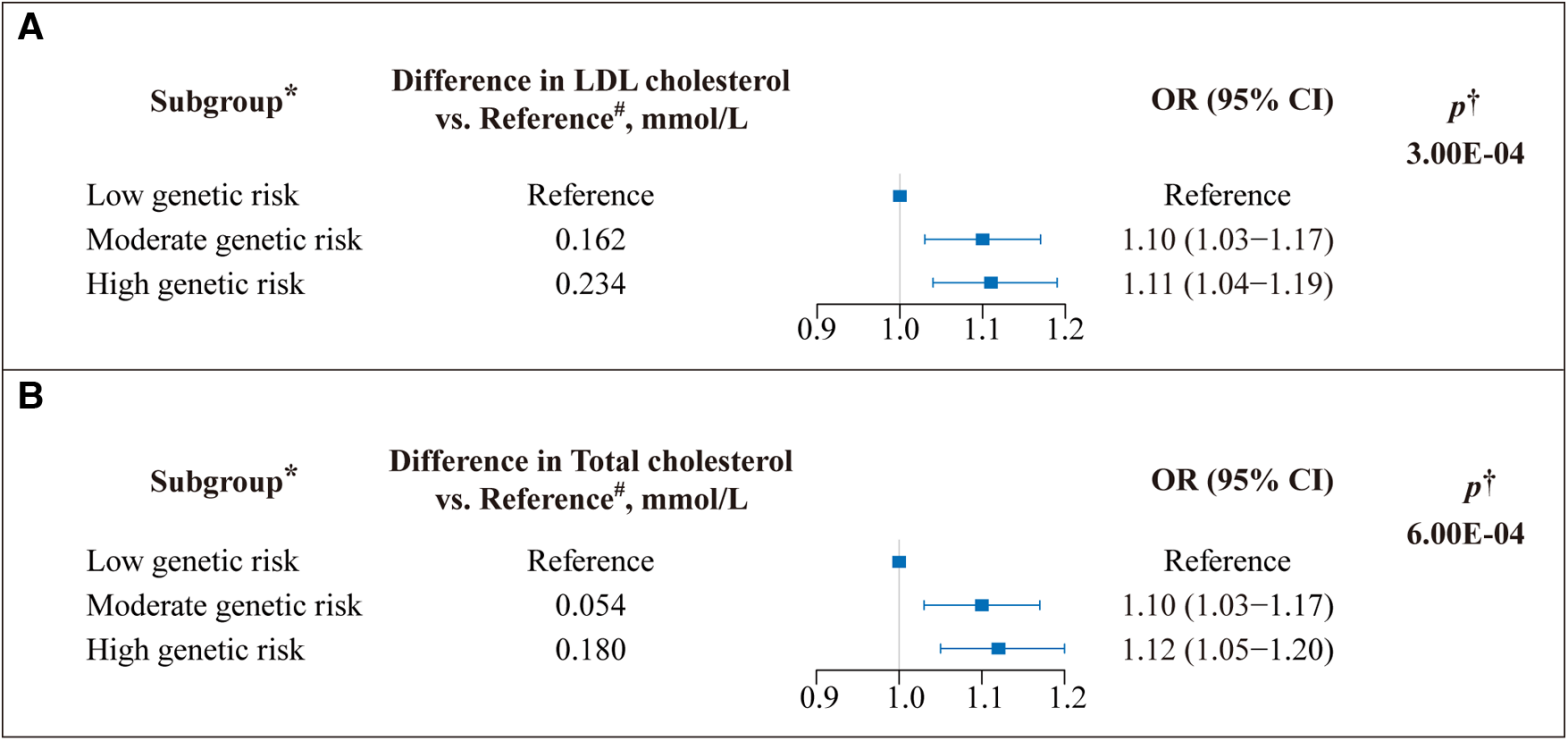
Effect of *VEGFA* genetic score on the risk of cardiovascular disease (CVD). A weighted genetic score was calculated for *VEGFA* for each study participant. (**A**) The genetic risk contributions to the CVD of SNPs on *VEGFA* associated with low-density lipoprotein (LDL) cholesterol and (**B**) total cholesterol (TC) (Unit: millimoles per liter) levels were calculated using the Ldpred2 method, respectively. OR: odds ratio; Boxes represent point estimates of effect. Lines represent 95% confidence intervals (CIs). *The genetic score was classified into three subgroups based on tertiles to determine genetic risk. A low genetic score indicates low genetic risk, and vice versa. ^#^Represents the difference between each group's mean lipid (LDL-C/TC) level and the mean lipid level of the reference group. ^†^Adjusted for age, sex, ethnicity, assessment center, Townsend index, alcohol frequency, smoke status, BMI, and cholesterol-lowering medicine.

GRS was calculated based on *VEGFA* variants to reflect LDL-C and TC levels. Subsequently, we assessed the relationship between GRS and cardiovascular risks. Compared with the high *VEGFA* score, the lower *VEGFA* score showed an 11% (OR = 1.11, 95% CI: 1.04–1.19) reduced risk of CVD, *p* = 3.00×10^−4^ (Figure 1A); likewise, the lower *VEGFA* score for TC was associated with a 12% (OR = 1.12, 95% CI: 1.05–1.20) decreased the risk of CVD, *p* = 6.00×10^−4^ (Figure 1B). A dose-response stratified analysis showed a stepwise decrease in LDL-C and TC levels (calculated based on *VEGFA* variants) with a corresponding decrease in the risk of CVD (Figures 1A,B).

### 3.4. One-Sample Mendelian randomization

We used GRS of *VEGFA* genetic variants as instrumental variables in one-sample MR analysis. In the first stage, the F statistics for instrumental variables (*VEGFA* variation) was 20.76 (*p* = 5.2×10^−6^), suggesting weak instrumental variable bias was avoided. In the second stage, the predicted LDL-C level produced from the first stage was significantly associated with the risk of CVD (OR = 1.06, 95% CI: 1.02–1.11, *p *= 8.4×10^−3^). These results together suggested that LDL-C level, determined by *VEGFA* genetic variants, was casually associated with CVD. However, since the GRS of *VEGFA* genetic variants was not associated (*p *> 0.05) with TC level, we could not infer the causal relationship between TC and CVD risk.

### 3.5. Sensitivity analysis

Sensitivity analyses confirmed the robustness of the observational findings. We reanalyzed the association between the five lipid levels and CVD by excluding participants who experienced a CVD event within two years of the initial follow-up. We observed no significant alterations in the results. Specifically, LDL-C, RC, and TG remained positively associated with the incidence of CVD, whereas HDL-C remained a protective factor against CVD (Supplementary Table 7).

## 4. Discussion

In the UK Biobank population, our investigation revealed a correlation between *VEGFA* variation and alternations in blood lipoprotein profiles. The increased risk of cardiovascular events could be attributed to the elevated LDL-C level reflected by the *VEGFA* genetic risk. These findings mimic the effects achieved by *VEGFA* inhibition in reducing LDL-C levels, underscoring the potential of *VEGFA* suppression as a viable genetic target for therapeutic interventions.

CVD is a multifaceted and heritable condition that results from a combination of genetic and environmental factors. Dyslipidemia, a common lipid abnormality, is an established risk factor for CVD. Several prospective cohort studies and meta-analyses have shown that maintaining optimal levels of LDL-C could reduce the lifetime risk of atherosclerosis, while high levels of HDL-C may protect against CVD (43–45). Our observational study unveiled associations between elevated LDL-C, RC, and TG levels with CVD. However, the relationship between TC and CVD remains uncertain. In alignment with our findings, other studies have demonstrated either a positive association (44, 46, 47) or a weak or nonexistent connection (48–50). Hence, it is crucial to perform additional research to enhance our comprehension of the factors that influence the prevention and susceptibility to CVD.

We developed the GRS based on genetic variants in *VEGFA* by utilizing GWAS summary data from the GLGC. Our GRS is designed to mimic the lifetime exposure of lipids. SNP association tests in GWAS are typically conducted one SNP at a time, leading to strong linkage disequilibrium (LD) across the genome and biased estimates of independent effect (51). We used the LDpred2 method to construct the *VEGFA* GRS to overcome this issue. LDpred2 is an updated and powerful tool that derives multi-gene scores based solely on summary statistics and LD matrices. Unlike other gene scoring methods that rely on marker pruning with LD and applying *p*-value thresholds to association statistics, LDpred2 retains more information to improve prediction accuracy (38) and solves the problem of model specification errors while improving computational efficiency. It has been used in assessing the risk of numerous diseases and has significantly enhanced the performance and reliability of risk prediction in recent years (52–55).

Stratifying populations by different *VEGFA* genetic risks, we have found that individuals with higher *VEGFA* genetic risk have an increased risk of CVD, which aligns with previous studies. Li et al. identified *VEGF* gene polymorphisms rs699947 and haplotypes as potential genetic markers for coronary heart disease pathogenesis (56). Another meta-analysis demonstrated that *VEGFA* rs699947 C>A, rs3025039 C>T, and rs2010963 G>C polymorphisms are risk factors for coronary heart disease (57). Animal studies have also shown that overexpression of *VEGFA* increases the likelihood of atherosclerosis in *ApoE*-deficient mice (26). Clinical research also supports controlling angiogenesis and *VEGFA* to improve the quality of life and life expectancy among cardiac patients (58). In addition, a marginal elevation in LDL-C levels was observed across low-, moderate- to high-genetic-risk subgroups; however, the clinical implications of this result remain uncertain. Firstly, owing to the multifactorial nature of CVD and the complexities of lipid profiles, neither LDL-C levels nor *VEGFA* variations act in isolation. This, in part, elucidates the rationale behind the marginal LDL-C increases observed in our study. Secondly, the clinical paradigm of LDL-C reduction has evolved as a cornerstone in CVD prevention and management. Recent guidelines for blood lipid management emphasize the regular monitoring of treatment efficacy and the surveillance of potential adverse reactions. These guidelines advocate flexible treatment plans to ensure sustained adherence to lipid standards. Furthermore, studies have established a correlation between cumulative LDL-C exposure, lipid-lowering treatment, and the risk of atherosclerotic cardiovascular disease (ASCVD) (59, 60). Therefore, from a collective perspective, meticulous attention should be directed toward achieving early, sustained, and stable attainment of LDL-C targets. Lastly, precision medicine seeks to tailor medical decisions and interventions to individual characteristics. Our findings, although showing marginal changes, may contribute to more precise risk prediction. In the context of precision medicine, identifying subgroups with even minor increases in LDL-C levels can help guide targeted interventions and preventive strategies.

Given *VEGFA*'s pivotal role in vascular angiogenesis across diverse physiological and pathological contexts, as well as its significant contribution to vascular homeostasis (61, 62), a previous study has revealed a robust association between common genetic variations linked to *VEGFA* and both HDL-C and LDL-C (30). Furthermore, Dabravolski et al. have proposed the potential of the *VEGF* family as a therapeutic target for atherosclerosis (63). Thus, by utilizing a lipid genetic score constructed from genetic variations associated with *VEGFA*, our study extended its inquiry, shedding light on a causal link between lipid metabolism and CVD risk. Previous research has firmly established the additional CVD risk associated with pathogenic FH variations (64–66). Currently, statins, ezetimibe, bile acid sequestrants, and *PCSK9* inhibitors are used as standard agents for lipid-lowering interventions. However, whether administered individually or in combination, these treatments often engender intolerable side effects, necessitating the exploration of novel drug targets and clinical trials (67). Mechanistic investigations into the interplay between *VEGFA* and lipid metabolism suggest that *VEGFA* may downregulate lipoprotein lipase activity, leading to the accumulation of TC within large lipoprotein particles, including chylomicrons and VLDL, thereby fostering atherogenic changes (26). Clinical studies further report that lipid levels in the bloodstream can modulate *VEGFA* expression and influence biological activity (68). In addition, molecular mechanistic studies have shown that elevated LDL level impairs angiogenesis via disrupting an endothelial cell-autonomous signaling network (*TNFα/NF-κB/HIF/VEGF*) that governs angiogenesis in hypoxic responses (69). This may represent a mechanistic link through which lipid levels impact the onset and progression of CVD. Our genetic findings underscore that *VEGFA* variations accounting for elevated LDL-C levels correlate with an increased risk of cardiovascular events, thus establishing a causal connection between LDL-C and CVD. This genetic evidence lends support to the proposition of *VEGFA* as a promising therapeutic target.

It is important to note that our study has several limitations. Firstly, despite extensive evidence of *VEGFA*'s role in lipid regulation and CVD treatment (60, 63, 70), there is currently a lack of effective agents capable of targeting *VEGFA* to modulate lipid levels in either experimental or clinical settings. Predicting or investigating the effects of genetic variants that mimic therapeutic actions and assessing their population-specific specificity can be inherently challenging. Secondly, in alignment with all MR studies, we must acknowledge our inability to validate our instrumental variable hypothesis empirically. Our estimates are susceptible to biases stemming from pleiotropy or confounding factors. Therefore, where conditions allow, it becomes imperative to bolster causal inferences through more robust randomized controlled trials. Thirdly, given the polygenic underpinnings of lipid expression profiles and CVD risk, our investigation concentrated solely on evaluating the impact of a single genetic variation within the *VEGFA* gene on CVD susceptibility. It is worth noting that while numerous studies in the field of lipid metabolism and its association with CVD have historically focused on variations in single genes, such as *PCSK9*, *HMGCR*, and *ACLY* (5, 71), *VEGFA* may only represent a single node in this complex network. Thus, further research endeavors aimed at elucidating interactions between *VEGFA* and other genes and exploring *VEGFA*'s integration into the broader polygenic context are warranted to bridge the gap between monogenic insights and polygenic complexities. Lastly, it is essential to acknowledge that, due to database limitations, the population included in the MR study only consisted of individuals from the UK Biobank. Subsequent research should expand data collection to encompass diverse populations, ensuring the generalizability of research findings.

In conclusion, within the UK Biobank population, we have observed a statistical association between LDL-C levels influenced by *VEGFA* variations and susceptibility to cardiovascular events. Our findings suggest the potential importance of *VEGFA* inhibition in addressing CVD caused by lipid dysregulation. While our discovery highlights *VEGFA* as a promising therapeutic target in cardiovascular research, it's essential to recognize that additional experimental and clinical validation is warranted to firmly establish its application and significance in CVD prevention and precision treatment.

## Data Availability

Publicly available datasets were analyzed in this study. This data can be found here: UK Biobank (https://www.ukbiobank.ac.uk); Global Lipids Genetics Consortium (http://csg.sph.umich.edu/willer/public/Lipids2013/).
