# Supplementary material for: Variation in VEGFA and risk of cardiovascular disease in the UK Biobank
Source: Front Cardiovasc Med. 2023 Nov 28;10:1240288. doi: 10.3389/fcvm.2023.1240288 (PMC10713833; doi:10.3389/fcvm.2023.1240288)
Supplement: Supplementary file 1 [file Datasheet1.docx]

Supplementary Material

**Variation in *VEGFA* and Risk of Cardiovascular Disease in the UK Biobank**

**Hongyin Chen^1†^, Xingyu Lv^1†^, Jinzhao Yang^1^, Zhaojun Chen^1^, Wanning Qiao^1^, Tao Zhou^1*^, Yang Zhang^1,2*^**

*** Correspondence:**

Yang Zhang：[zhangy2293@mail.sysu.edu.cn](mailto:zhangy2293@mail.sysu.edu.cn)

Tao Zhou：[zhout93@mail.sysu.edu.cn](mailto:zhout93@mail.sysu.edu.cn)

# Supplementary Tables and Figures

# Supplementary Tables

**Supplementary Table 1**. The primary analysis of genetic variants in *VEGFA* associated with high-density lipoprotein-cholesterol (HDL-C) Level

| SNP | Position | EA/OA | EAF | beta | *p-*value |
| --- | --- | --- | --- | --- | --- |
| rs186369938 | 6:43737983 | C | 0.025 | -0.0262 | 1.74E-06 |
| rs3024994 | 6:43743507 | T | 0.0495 | 0.0152 | 4.13E-05 |
| rs3025032 | 6:43751037 | T | 0.333 | 0.00694 | 0.00016 |
| rs3025039 | 6:43752536 | T | 0.137 | 0.00932 | 6.46E-05 |
| rs3025053 | 6:43753325 | A | 0.104 | 0.01041 | 0.00015 |
| rs833061 | 6:43737486 | C | 0.487 | 0.00211 | 0.205 |
| rs943070 | 6:43740451 | G | 0.184 | -0.0002 | 0.919 |

**Supplementary Table 2**. The primary analysis of genetic variants in *VEGFA* associated with low-density lipoprotein-cholesterol (LDL-C) Level

| SNP | Position | EA/OA | EAF | beta | *p-*value |
| --- | --- | --- | --- | --- | --- |
| rs186369938 | 6:43737983 | C | 0.0249 | -0.00651307 | 2.49E-01 |
| rs3024994 | 6:43743507 | T | 0.0496 | -0.0116051 | 2.32E-03 |
| rs3025032 | 6:43751037 | T | 0.331 | 0.00131703 | 0.484 |
| rs3025039 | 6:43752536 | T | 0.137 | -0.00662901 | 5.61E-03 |
| rs3025053 | 6:43753325 | A | 0.105 | -0.00948014 | 0.000811 |
| rs833061 | 6:43737486 | C | 0.487 | 0.00308584 | 0.0712 |
| rs943070 | 6:43740451 | G | 0.185 | -0.00530058 | 0.0136 |

**Supplementary Table 3**. The primary analysis of genetic variants in *VEGFA* associated with triglycerides (TG) Level

| SNP | Position | EA/OA | EAF | beta | *p*-value |
| --- | --- | --- | --- | --- | --- |
| rs186369938 | 6:43737983 | C | 0.025 | 0.0102 | 0.065 |
| rs3024994 | 6:43743507 | T | 0.0497 | -0.0225 | 1.86E-09 |
| rs3025032 | 6:43751037 | T | 0.331 | -0.0043 | 0.0202 |
| rs3025039 | 6:43752536 | T | 0.137 | -0.0172 | 3.49E-13 |
| rs3025053 | 6:43753325 | A | 0.104 | -0.0116 | 3.13E-05 |
| rs833061 | 6:43737486 | C | 0.486 | 0.00154 | 0.361 |
| rs943070 | 6:43740451 | G | 0.185 | -0.0002 | 0.924 |

**Supplementary Table 4**. The primary analysis of genetic variants in *VEGFA* associated with total cholesterol (TC) Level

| SNP | Position | EA/OA | EAF | beta | *p*-value |
| --- | --- | --- | --- | --- | --- |
| rs186369938 | 6:43737983 | C | 0.0251 | -0.0072 | 0.182 |
| rs3024994 | 6:43743507 | T | 0.0494 | -0.0115 | 0.00155 |
| rs3025032 | 6:43751037 | T | 0.332 | 0.00145 | 0.42 |
| rs3025039 | 6:43752536 | T | 0.137 | -0.0101 | 9.69E-06 |
| rs3025053 | 6:43753325 | A | 0.104 | -0.0097 | 0.00033 |
| rs833061 | 6:43737486 | C | 0.487 | 0.00369 | 0.0237 |
| rs943070 | 6:43740451 | G | 0.184 | -0.0041 | 0.0472 |

**Supplementary Table 5**. The primary analysis of genetic variants in *VEGFA* associated with remnant cholesterol (RC) Level

| SNP | Position | EA/OA | EAF | beta | *p*-value |
| --- | --- | --- | --- | --- | --- |
| rs186369938 | 6:43737983 | C | 0.028 | 0.00728 | 0.281 |
| rs3024994 | 6:43743507 | T | 0.0501 | -0.0191 | 4.60E-05 |
| rs3025032 | 6:43751037 | T | 0.333 | -0.0024 | 0.321 |
| rs3025039 | 6:43752536 | T | 0.135 | -0.0129 | 2.01E-05 |
| rs3025053 | 6:43753325 | A | 0.102 | -0.0154 | 7.91E-06 |
| rs833061 | 6:43737486 | C | 0.482 | 0.00302 | 0.165 |
| rs943070 | 6:43740451 | G | 0.186 | -0.0023 | 0.391 |

**Supplementary Table 6.** Association results between CVD and phenotypic lipid profiles after adjusting variants in *LDLR, APOB,* and *PCSK9*

|  | **Adjusted demographics^a^** | | ***p*** | ***p._adjusted_*^b^** |
| --- | --- | --- | --- | --- |
|  | **OR** | **95%CI** |  |  |
| LDL-C | 1.09 | 1.06-1.11 | 1.5E-12 | 7.50E-12 |
| HDL-C | 0.81 | 0.76-0.86 | 3.4E-11 | 8.50E-11 |
| RC | 1.24 | 1.13-1.37 | 7.5E-06 | 1.25E-05 |
| TC | 1.05 | 1.00-1.09 | 0.036 | 3.60E-02 |
| TG | 1.15 | 1.08-1.22 | 1.6E-05 | 2.00E-05 |

LDL-C, Low-density lipoprotein-cholesterol; HDL-C, High-density lipoprotein-cholesterol; RC, Remnant cholesterol; TC, Total cholesterol; TG, Triglycerides; OR, Odds ratio; Cl, Confidence Interval.

^a^Adjusted for age, sex, ethnicity, assessment center, Townsend index, alcohol frequency, smoke status, BMI, cholesterol-lowering medicine, and familial hypercholesterolemic variants in *LDLR, APOB,* and *PCSK9* genes.

^b^The adjusted *p* was calculated by controlling the *BH*.

**Supplementary Table 7.** Sensitivity Analysis

|  | **Adjusted demographics^a^** | | ***p*** | ***p._adjusted_*^b^** |
| --- | --- | --- | --- | --- |
|  | **OR** | **95%CI** |  |  |
| LDL-C | 1.08 | 1.06-1.11 | 2.4E-10 | 1.2E-09 |
| HDL-C | 0.82 | 0.77-0.88 | 1.4E-08 | 3.5E-08 |
| RC | 1.26 | 1.14-1.40 | 7.0E-06 | 1.17E-05 |
| TC | 1.06 | 1.01-1.11 | 0.02 | 0.02 |
| TG | 1.16 | 1.09-1.25 | 1.1E-05 | 1.38E-05 |

LDL-C, Low-density lipoprotein-cholesterol; HDL-C, High-density lipoprotein-cholesterol; RC, Remnant cholesterol; TC, Total cholesterol; TG, Triglycerides; OR, Odds ratio; Cl, Confidence Interval.

^a^Adjusted for age, sex, ethnicity, assessment center, Townsend index, alcohol frequency, smoke status, BMI, cholesterol-lowering medicine.

^b^The adjusted *p* was calculated by controlling the *BH*.

# Supplementary Figures


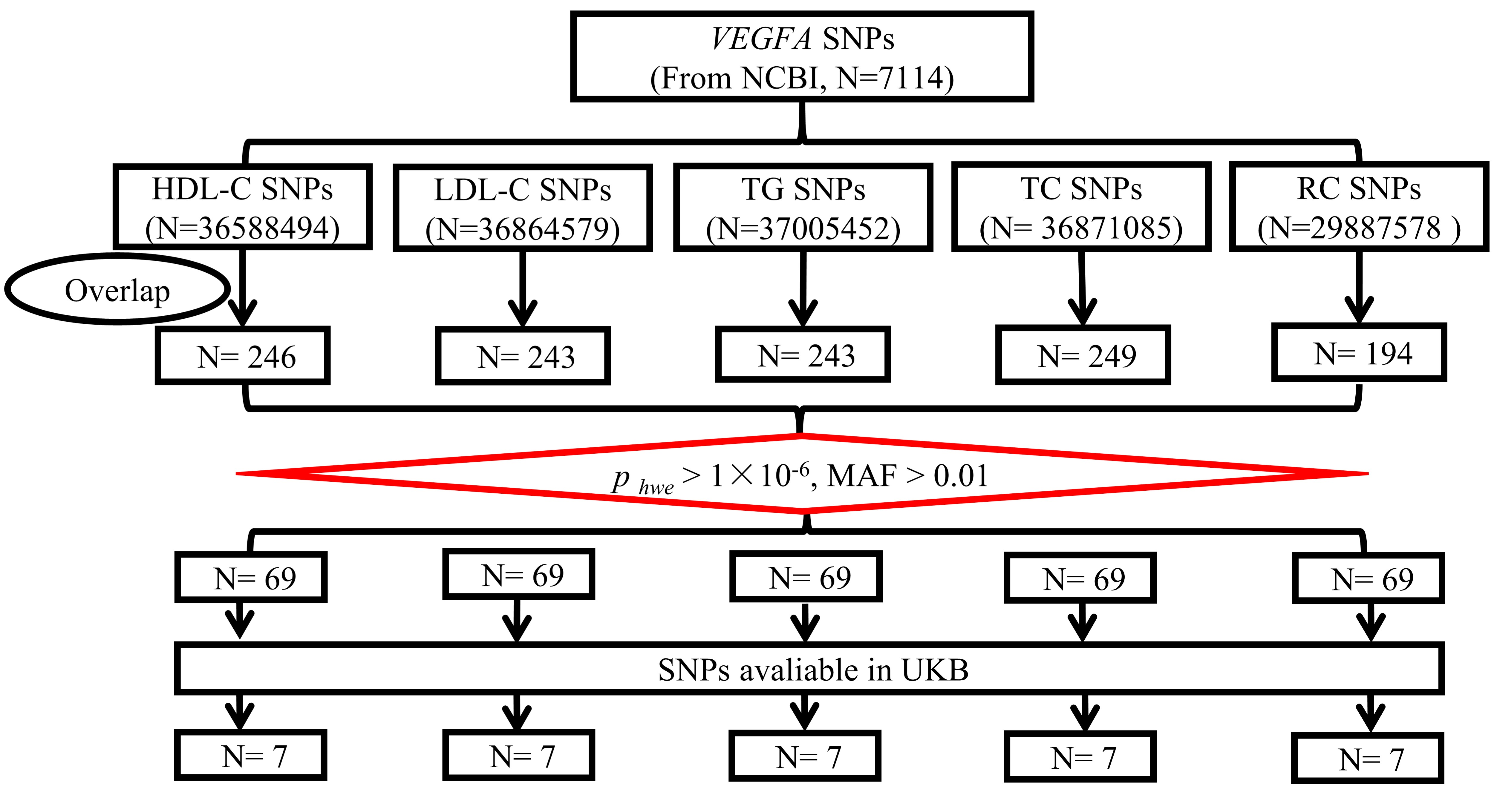


**Supplementary Figure 1**. Flow chart of *VEGFA* SNP screening for inclusion of 5 lipids gene risk scores. We obtained 7114 SNPs of the *VEGFA* gene from the NCBI SNP database (https://www.ncbi.nlm.nih.gov/snp). The above SNPs were overlapped with the SNPs in HDL-C, LDL-C, TC, TC and RC reported by the Global Lipids Genetics Consortium (GLGC: http://csg.sph.umich.edu/willer/public/ lipids 2013/) to obtain the SNP sets of five lipids. Then, we retained SNPs with a Hardy-Weinberg Equilibrium (HWE) *p*-value (*p_hwe_*) > 1×10^-6^ and minor allele frequency (MAF) > 0.01. Next, SNPs (n=7) were matched to the levels of the above five lipids in the UKB database, and finally, these SNPs were included in the gene scores of the five lipids. HDL-C, High-density lipoprotein-cholesterol; LDL-C, low-density lipoprotein-cholesterol; TG, Triglycerides; TC, total cholesterol; RC, Remnant cholesterol.


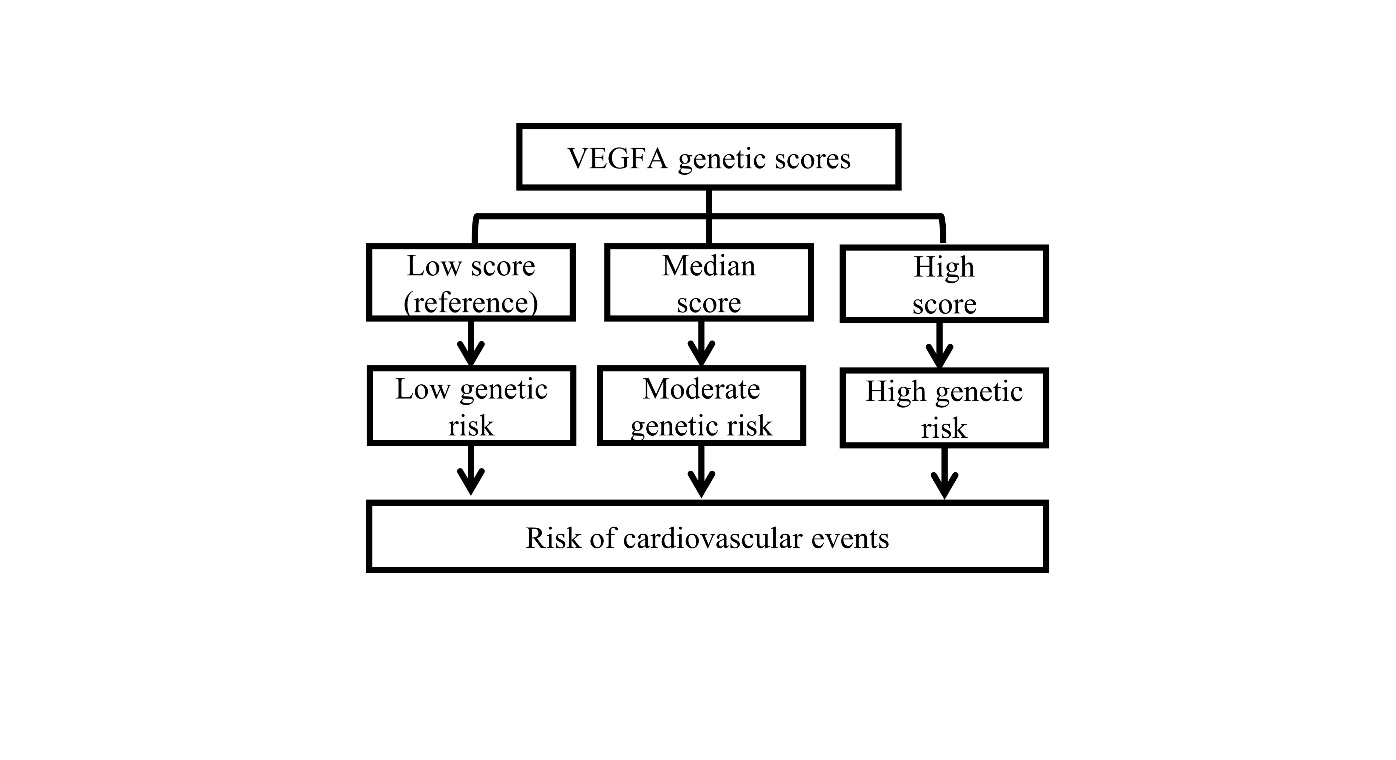


**Supplementary Figure 2**. Experimental design and grouping. We divided the genetic score of *VEGFA* into three groups according to tertiles, and the genetic risk can be classified as low, moderate, or high based on the score accordingly. The risk of cardiovascular events was compared in these three groups.


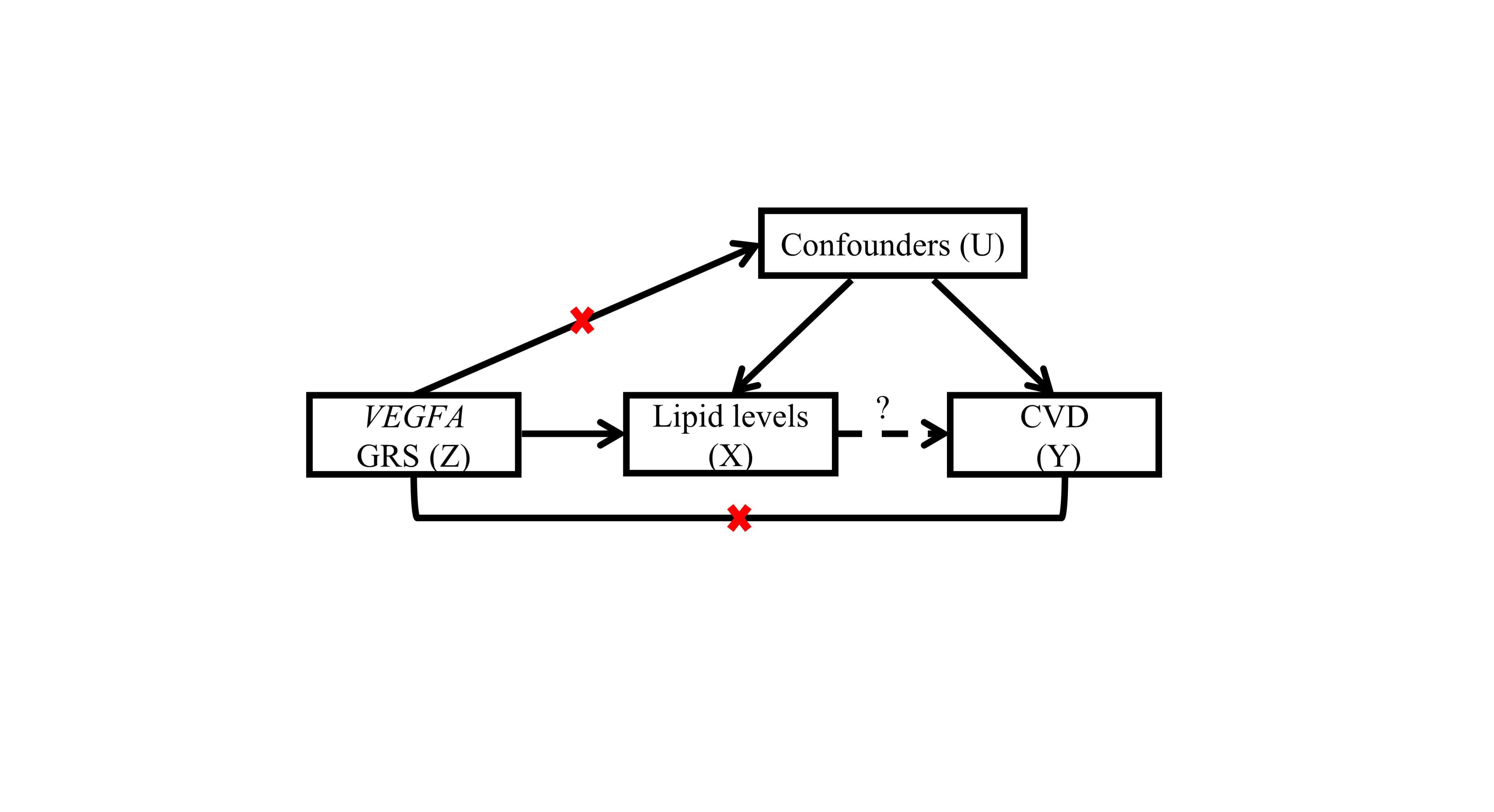


**Supplementary Figure 3**. MR framework. Z: *VEGFA* genetic score; X: lipids exposure level (Unit: mmol/L); Y: cardiovascular disease (CVD) risk; U: Confounders include age, sex, ethnicity, BMI, cholesterol-lowering medicine, genotyping batch, and PC1-PC10.
